# Posttranscriptional Suppression of Lipopolysaccharide-Stimulated Inflammatory Responses by Macrophages in Middle-Aged Mice: A Possible Role for Eukaryotic Initiation Factor 2**α**

**DOI:** 10.1155/2014/292986

**Published:** 2014-04-07

**Authors:** Ken Shirato, Kazuhiko Imaizumi

**Affiliations:** ^1^Laboratory of Physiological Sciences, Faculty of Human Sciences, Waseda University, 2-579-15 Mikajima, Tokorozawa, Saitama 359-1192, Japan; ^2^Global Center of Excellence Doctoral Program, Graduate School of Sport Sciences, Waseda University, 2-579-15 Mikajima, Tokorozawa, Saitama 359-1192, Japan

## Abstract

The intensities of macrophage inflammatory responses to bacterial components gradually decrease with age. Given that a reduced rate of protein synthesis is a common age-related biochemical change, which is partially mediated by increased phosphorylation of eukaryotic initiation factor-2**α** (eIF-2**α**), we investigated the mechanism responsible for the deterioration of macrophage inflammatory responses, focusing specifically on the age-related biochemical changes in middle-aged mice. Peritoneal macrophages isolated from 2-month-old (young) and 12-month-old (middle-aged) male BALB/c mice were stimulated with lipopolysaccharide (LPS). Although LPS-stimulated secretion of tumor necrosis factor-**α** (TNF-**α**) by the macrophages from middle-aged mice was significantly lower than that from young mice, LPS caused marked increases in levels of TNF-**α** mRNA in macrophages from middle-aged as well as young mice. Moreover, LPS evoked similar levels of phosphorylation of c-Jun N-terminal kinase (JNK) and nuclear factor-**κ**B (NF-**κ**B) in young and middle-aged mice. In contrast, the basal level of phosphorylated eIF-2**α** in macrophages from middle-aged mice was higher than that in macrophages from young mice. Salubrinal, an inhibitor of the phosphatase activity that dephosphorylates eIF-2**α**, suppressed the LPS-stimulated inflammatory responses in a murine macrophage cell line RAW264.7. These results suggest that posttranscriptional suppression of macrophage inflammatory responses during middle age requires phosphorylation of eIF-2**α**.

## 1. Introduction

Increased susceptibility to infectious diseases is a hallmark of advancing age and is associated with reduced functions of a variety of immune cells [1–6]. Among these cells are macrophages, which play critical roles in the first line of host defense against pathogenic microorganisms [1–6]. Macrophages use toll-like receptor (TLR) isoforms on their cell surfaces to recognize various pathogen components, such as the lipopolysaccharide (LPS); subsequent activation of downstream intracellular signaling leads to the production and secretion of proinflammatory mediators, including tumor necrosis factor-*α* (TNF-*α*) [7]. The acute local inflammation facilitates accumulation and activation of immune cells in the infectious foci.

The level of production of LPS-induced inflammatory-promoting cytokines by macrophages decreases gradually with aging [8, 9]. Previous findings suggest defects of the intracellular signal transduction downstream of TLR in advanced-aged (18–24-month-old) mice. For instance, the cell surfaces of peritoneal and splenic macrophages from advanced-aged mice show reduced levels of the TLR4 coreceptor CD14 [9, 10]. Advanced-aged mice also show reduced levels of intracellular signaling proteins, such as mitogen-activated protein kinase (MAPK) and c-Jun N-terminal kinase (JNK), compared with younger mice [11, 12]. In addition, DNA microarray analysis indicated suppression of genes of the TLR signaling pathway responsible for nuclear factor-*κ*B (NF-*κ*B) activation in advanced-aged mice [13]. However, the mechanisms responsible for the attenuation of macrophage inflammatory responses in middle-aged mice have not been fully elucidated.

A decreased rate of bulk protein synthesis is one of the most common age-related biochemical changes [14, 15]. Translation of mRNA into protein is initiated by physical interactions of the methionyl tRNA specialized for initiation (Met-tRNAi), several eukaryotic initiation factor (eIF) subunits, and the 40S ribosome [16]. Of the eIF subunits, phosphorylation at Ser51 of eIF-2*α* competitively inhibits the GDP/GTP exchange catalytic activity of eIF-2B; this suppresses further binding of the Met-tRNAi complex with mRNA and subsequent protein synthesis [17, 18]. Compared with young mice, advanced-aged mice express higher levels of phosphorylated eIF-2*α* and its kinase, double-stranded RNA-dependent protein kinase (PKR), in the liver and kidney [19]. Levels of phosphorylated eIF-2*α* in the cerebral cortex of middle-aged mice are higher than those in young mice [20]. In contrast, there is a report that phosphorylation of a wide variety of eIF subunits, including eIF-2*α*, is reduced during aging in different rat tissues [21]. However, it is still unclear how advancing age affects phosphorylation of eIF-2*α* in macrophages and what roles eIF-2*α* plays in LPS-stimulated macrophage inflammatory responses.

In order to better understand the molecular mechanisms responsible for age-related deterioration of macrophage inflammatory responses, we examined LPS-stimulated production of TNF-*α* at the protein and mRNA levels; LPS-stimulated phosphorylation of JNK, NF-*κ*B, and inhibitor of *κ*B*α* (I*κ*B*α*); and the levels of phosphorylated forms of eIF-2*α* and PKR in resident peritoneal macrophages from 2-month-old (young) and 12-month-old (middle-aged) male BALB/c mice. Moreover, to elucidate a functional role of the inactive form of eIF-2*α*, we examined the effects of salubrinal, a selective inhibitor of phosphatase specific to eIF-2*α* [22–24], on the LPS-stimulated inflammatory responses in the murine macrophage cell line RAW264.7.

## 2. Materials and Methods

### 2.1. Mice

Two-month-old (young) and 12-month-old (middle-aged) male BALB/c mice (*n* = 12 for each age group) were purchased from Charles River Laboratories (Kanagawa, Japan) and prefed for a week to allow for adaptation to their new environment. The mice were housed individually in plastic cages at a temperature of 23–25°C and a relative humidity of 50–60% with a fixed light/dark cycle (light from 07:00 to 19:00 h and darkness from 19:00 to 07:00 h) [25, 26]. Food (CE-2 cubic type; CLEA Japan, Tokyo, Japan) and once-boiled tap water were available* ad libitum* [25, 26]. All animal care and experimental procedures were approved by the Committee of Animal Care, Ethics, and Use at Waseda University (number 2013-A031) and performed in accordance with the Guiding Principles for the Care and Use of Animals approved by the Council of the Physiological Society of Japan, based upon the Declaration of Helsinki of 1964.

### 2.2. Cell Preparation and Culture

After the adaptation period, peritoneal cells were harvested from the mice by sterile lavage of the peritoneal cavity with ice-cold Dulbecco's phosphate-buffered saline (DPBS; Nissui Pharmaceutical, Tokyo, Japan) [27–29]. The cells were resuspended in RPMI1640 medium (Sigma-Aldrich, St. Louis, MO, USA) supplemented with 2% heat-inactivated fetal bovine serum (FBS; BioWest, Nuaillé, France), 100 units/mL penicillin, and 100 mg/mL streptomycin (Nacalai Tesque, Kyoto, Japan). The cells from four mice were pooled, because the number of resident macrophages in the peritoneal cavity of a single mouse was insufficient for the experiments [27–29]. The cell density was adjusted to 2 × 10^6^ cells/mL, and the peritoneal cells were cultured at 37°C in a humidified incubator, containing 5% CO_2_ in the air, for 2 h to allow macrophages to adhere to the surface of the cell culture plate [25–29]. After nonadherent cells were removed, the cells were cultured with or without 100 ng/mL LPS from* Escherichia coli* (*E. coli*) 055:B5 (Sigma-Aldrich) for 6 h.

### 2.3. Enzyme-Linked Immunosorbent Assay (ELISA)

The cell culture supernatants were collected, centrifuged at 300 ×g for 5 min, and stored at −80°C for later use. The concentrations of TNF-*α* in the supernatants were measured using Quantikine ELISA Mouse TNF-*α* Immunoassay (R&D Systems, Minneapolis, MN, USA).

### 2.4. Reverse Transcriptase-Polymerase Chain Reaction (RT-PCR)

Total cellular RNA was extracted and purified from the cells using RNAiso Plus (Takara Bio, Shiga, Japan). One microgram of the total cellular RNA was converted to single-stranded cDNA using the Transcriptor First Strand cDNA Synthesis Kit (Roche Applied Science, Indianapolis, IN, USA). The cDNA (1 *μ*L) was subjected to PCR using Ex Taq DNA Polymerase (Takara Bio) and amplified by the following steps: denaturation at 94°C for 30 sec, annealing at 55°C for 30 sec, and then extension at 72°C for 30 sec. The primers used in this study are listed in Table 1. The PCR products were electrophoresed in 1.5% agarose gels that contained 1 *μ*g/mL ethidium bromide and then were visualized and quantitated using a LAS-3000 luminescent image analyzer (Fujifilm, Tokyo, Japan).

### 2.5. Western Blotting

The cells were washed three times with DPBS (Nissui), and then whole cellular protein was extracted with lysis buffer that contained 10 mM Tris HCl (pH 7.8), 1% Nonidet P-40, 0.15 M NaCl, and 1 mM ethylenediaminetetraacetic acid (EDTA) supplemented with EDTA-free Complete Protease Inhibitor Cocktail Tablet (Roche Applied Science) and EDTA-free Phosphatase Inhibitor Cocktail (Nacalai Tesque). Total cellular protein (10 *μ*g) was incubated at 37°C for 1 h in sample buffer, containing 75 mM Tris HCl (pH 6.8), 0.6% sodium dodecyl sulfate (SDS), 15% glycerol, 7.5% *β*-mercaptoethanol, and 9 *μ*g/mL bromophenol blue and then was separated by electrophoresis through 8% or 10% SDS-polyacrylamide gels. After transferring to polyvinylidene difluoride membrane (GE Healthcare, Buckinghamshire, UK), the membrane was blocked with 5% bovine serum albumin. Primary antibody against *β*-actin, phospho-JNK, JNK, phospho-p65, p65, phospho-I*κ*B*α*, I*κ*B*α*, phospho-eIF-2*α*, eIF-2*α*, glyceraldehyde-3-phosphate dehydrogenase (GAPDH), protein kinase RNA-like endoplasmic reticulum kinase (PERK) (Cell Signaling Technology, Danvers, MA, USA), phospho-PKR, or PKR (Santa Cruz Biotechnology, Dallas, TX, USA) was applied at a 1/1,000 dilution. Secondary antibody (horseradish peroxidase- (HRP-) conjugated AffiniPure Goat Anti-Rabbit IgG; Jackson Immuno Research Laboratories, West Grove, PA, USA) was applied at a 1/5,000 dilution. The membrane was incubated with Clarity Western ECL Substrate (Bio-Rad Laboratories, Hercules, CA, USA), and the target proteins were then visualized and quantitated using a LAS-3000 luminescent image analyzer (Fujifilm).

### 2.6. Cell Line and Culture

The murine macrophage cell line RAW264.7 was obtained from American Type Culture Collection (Manassas, VA, USA) and cultured in Dulbecco's Modified Eagle's medium (Sigma-Aldrich) supplemented with 10% heat-inactivated FBS (BioWest), 100 units/mL penicillin, and 100 mg/mL streptomycin (Nacalai Tesque). The culture conditions used are those described above. The cells were stimulated with 100 ng/mL LPS in the presence of 10 *μ*M salubrinal (Calbiochem, San Diego, CA, USA) or dimethyl sulfoxide (DMSO) alone for 6 h. The assays for the cell culture supernatants and cellular mRNA and proteins were performed as described above.

### 2.7. Statistical Analysis

Experimental data are presented as the mean ± standard error of the mean (SEM). Equivalence of group means was tested by one-way analysis of variance. Post hoc comparisons to determine significant differences among three groups or more were conducted using the Bonferroni test. Differences were considered significant when *P* < 0.05.

## 3. Results

### 3.1. Posttranscriptional Suppression of LPS-Stimulated Production of TNF-*α* by Peritoneal Macrophages from Middle-Aged Mice

**Figure 1:**
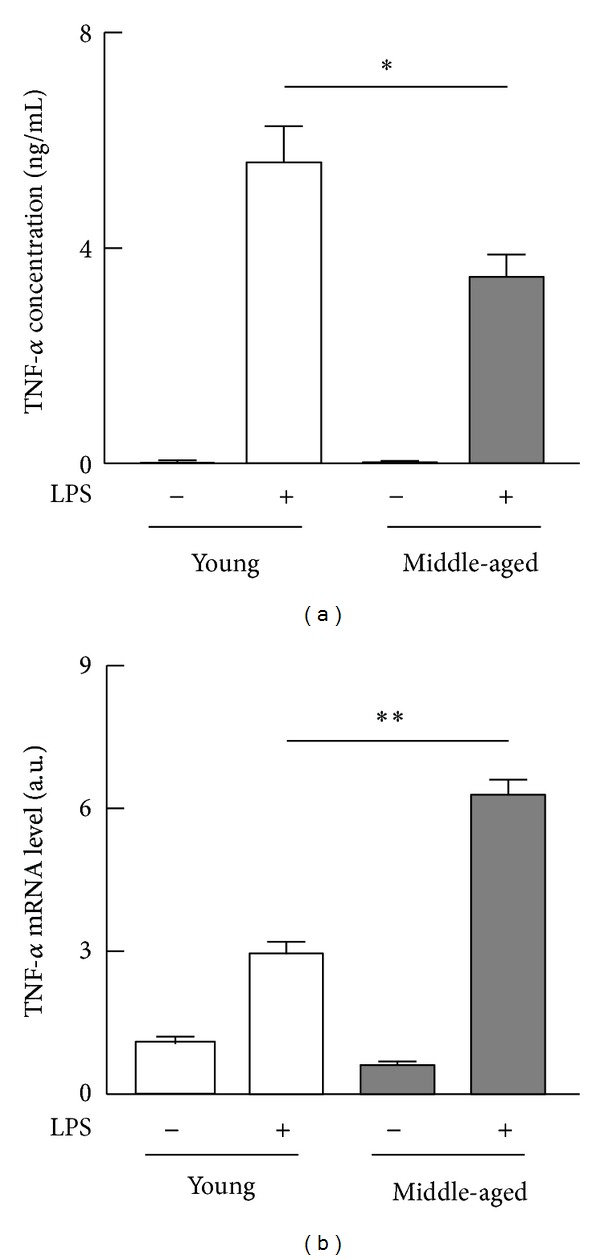
LPS-stimulated production of TNF-*α* by peritoneal macrophages from young and middle-aged mice. Resident peritoneal macrophages (pooled cells from four mice per each age group) from young or middle-aged mice were cultured with or without 100 ng/mL LPS for 6 h. The experiment was independently performed three times. (a) The concentrations of TNF-*α* in the culture supernatants were measured by ELISA. Values: means ± SEM (*n* = 3). *P < 0.05. (b) Total RNA extracted from macrophages was converted to cDNA to determine the levels of TNF-*α* mRNA by PCR. The values are corrected with the levels of GAPDH mRNA and 18S rRNA. Values: means ± SEM (*n* = 3). **P < 0.01.

We first examined LPS-stimulated production of TNF-*α* by peritoneal macrophages isolated from young and middle-aged mice. The concentrations of TNF-*α* in the culture supernatants of LPS-stimulated cells from middle-aged mice were significantly lower than those from young mice (Figure 1(a)). However, LPS caused a significantly higher increase in the level of* TNF-*α** mRNA in the cells from middle-aged mice than in the cells from young mice (Figure 1(b)). These results suggest that the onset of middle age is associated with posttranscriptional suppression of LPS-stimulated production of TNF-*α* by peritoneal macrophages.

### 3.2. Activation of JNK and NF-*κ*B Signaling by LPS in Peritoneal Macrophages from Middle-Aged Mice

**Figure 2:**
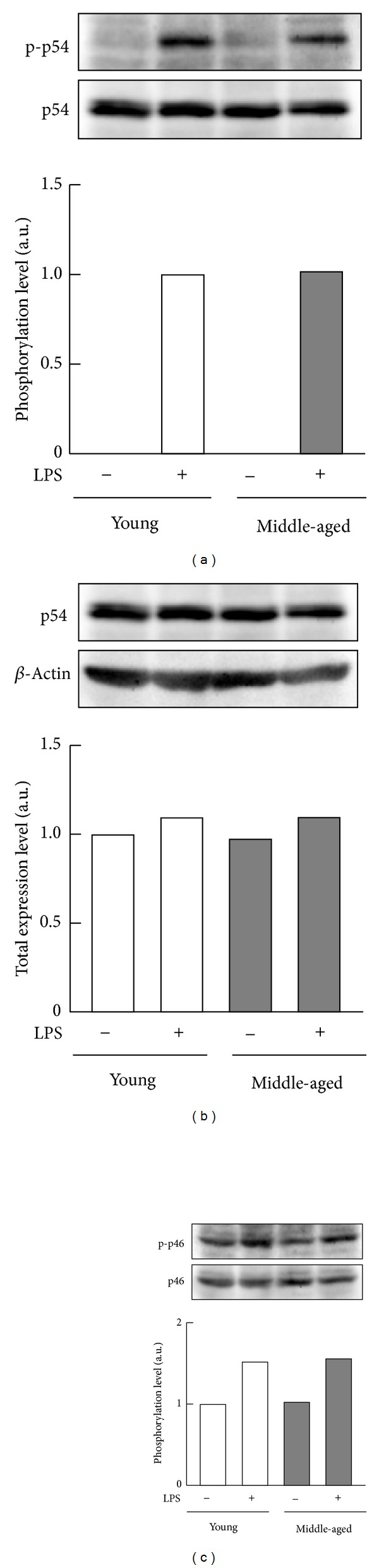
LPS-stimulated JNK phosphorylation of peritoneal macrophages from young and middle-aged mice. Resident peritoneal macrophages (pooled cells from four mice per each age group) from young or middle-aged mice were cultured with or without 100 ng/mL LPS for 6 h. (a) Phosphorylation of the p54 subunit of JNK, (b) abundances of the p54 subunit of JNK, and (c) phosphorylation of the p46 subunit of JNK were analyzed by Western blotting. Values are shown as means from duplicate samples.

**Figure 3:**
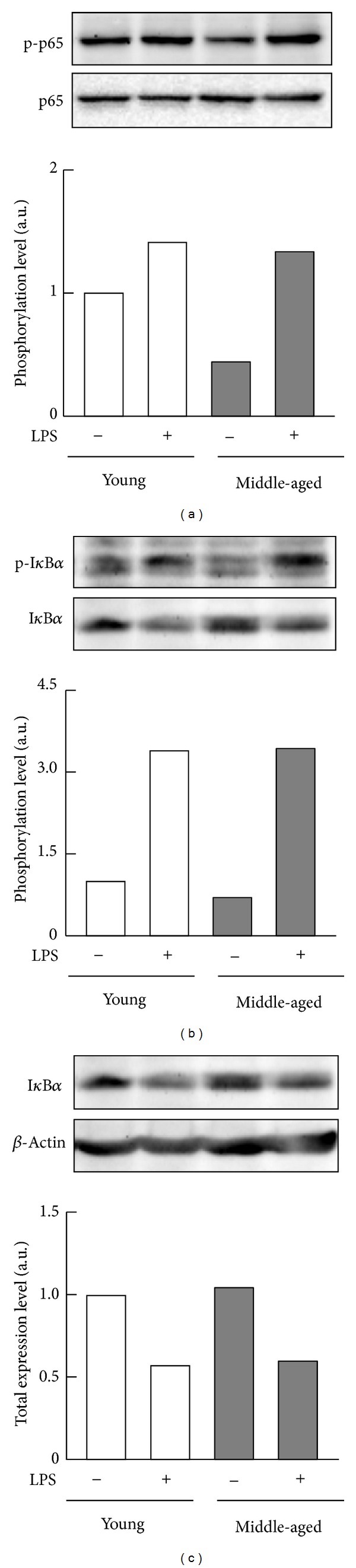
LPS-stimulated NF-*κ*B phosphorylation of peritoneal macrophages from young and middle-aged mice. Resident peritoneal macrophages (pooled cells from four mice per each age group) from young or middle-aged mice were cultured with or without 100 ng/mL LPS for 6 h. (a) Phosphorylation of the p65 subunit of NF-*κ*B, (b) phosphorylation of I*κ*B*α*, and (c) abundances of I*κ*B*α* were analyzed by Western blotting. Values are shown as means from duplicate samples.

To verify transduction of the signal initiated by the LPS receptor TLR4 in macrophages from middle-aged mice, we next compared LPS-stimulated phosphorylation of the p54 and p46 subunits of JNK, the p65 subunit of NF-*κ*B, and the I*κ*B*α* protein in macrophages from young and middle-aged mice. Macrophages from middle-aged as well as young mice evoked similar levels of phosphorylation of the JNK p54 subunit in response to LPS (Figure 2(a)), and total expression levels were comparable for macrophages collected for mice of both age groups (Figure 2(b)). Exposure to LPS caused comparable increases in the levels of phosphorylation of the p46 subunit of JNK in macrophages from young and middle-aged mice (Figure 2(c)). Although the level of phosphorylated p65 in the cells from middle-aged mice was lower than that from young mice in the absence of stimulation by LPS, levels of phosphorylated p65 increased to the same extent in the two groups of samples after exposure to LPS (Figure 3(a)). In addition, LPS stimulated comparable levels of phosphorylation and degradation of I*κ*B*α* in macrophages from either young or middle-aged mice (Figures 3(b) and 3(c)). These results indicate normal activation of JNK and NF-*κ*B signaling by LPS in peritoneal macrophages from middle-aged mice.

### 3.3. Peritoneal Macrophages from Middle-Aged Mice Have Higher Levels of eIF-2*α* Phosphorylation than Those from Young Mice

**Figure 4:**
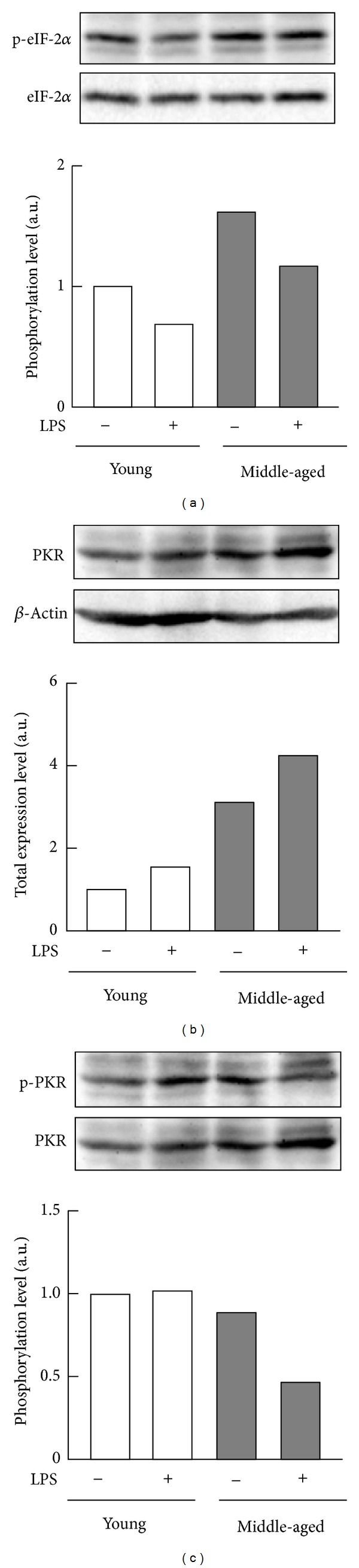
Phosphorylation of eIF-2*α* and PKR in peritoneal macrophages from young and middle-aged mice. Resident peritoneal macrophages (pooled cells from four mice per each age group) from young or middle-aged mice were cultured with or without 100 ng/mL LPS for 6 h. (a) Phosphorylation of eIF-2*α*, (b) abundances of PKR, and (c) phosphorylation of PKR were analyzed by Western blotting. Values are shown as means from duplicate samples.

**Figure 5:**
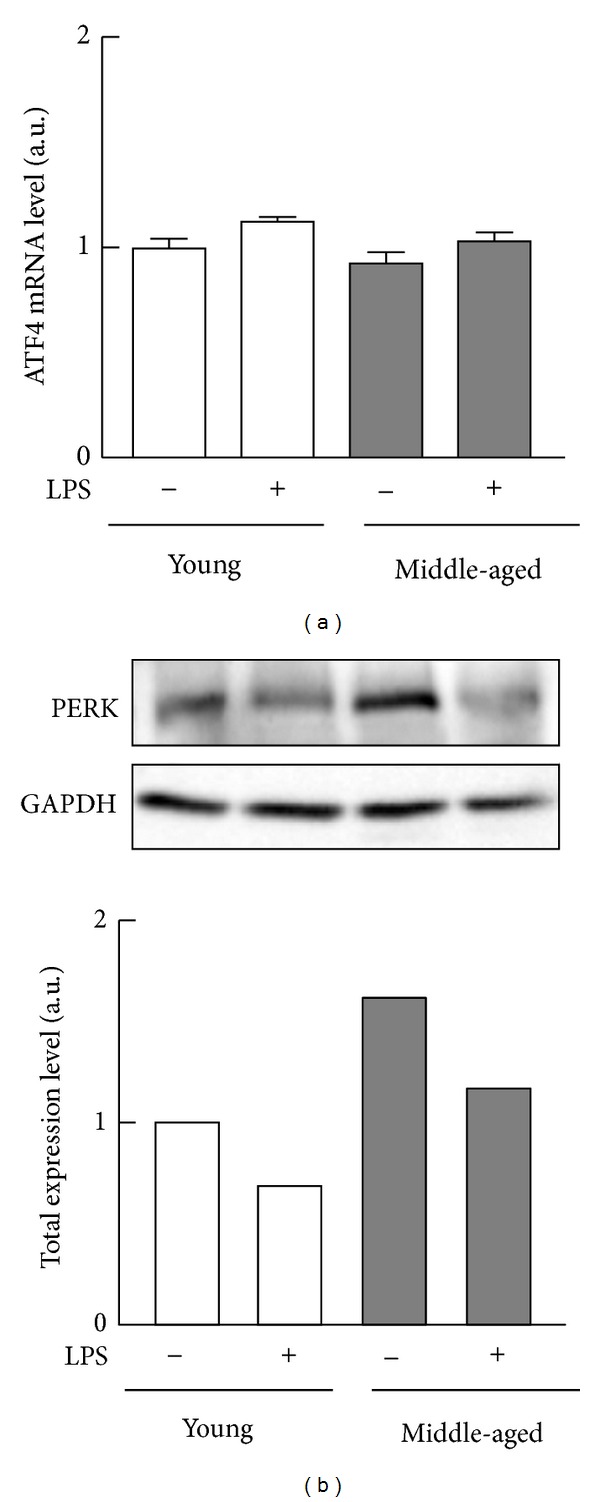
Expressions of ATF4 mRNA and PERK protein in peritoneal macrophages from young and middle-aged mice. Resident peritoneal macrophages (pooled cells from four mice per each age group) from young or middle-aged mice were cultured with or without 100 ng/mL LPS for 6 h. (a) Total RNA extracted from macrophages was converted to cDNA to determine the levels of ATF4 mRNA by PCR. The values are corrected with the levels of GAPDH mRNA and 18S rRNA. Values: means ± SEM (*n* = 3). (b) Abundances of PERK were analyzed by Western blotting.

Given that phosphorylation at Ser51 of eIF-2*α* reduces rates of protein translation [17, 18], we next compared the expression levels of the phosphorylated form of eIF-2*α* and its kinase (PKR) in the macrophages from young and middle-aged mice. In the absence of LPS, phosphorylation at Ser51 of eIF-2*α* in the cells from middle-aged mice was higher than that from young mice (Figure 4(a)). Exposure to LPS decreased the levels of phosphorylation at Ser51 in both groups of macrophages, although the level of phosphorylation in macrophages from middle-aged mice was higher than that for macrophages from young mice (Figure 4(a)). However, there were no significant differences in the mRNA expression level of* ATF1*, the target gene of eIF-2*α*, between middle-aged and young mice (Figure 5(a)). Moreover, macrophages from middle-aged mice contained more PKR than those from young mice in the absence of LPS (Figure 4(b)), and levels of PKR phosphorylation in macrophages from both groups of mice were consistent with the levels of PKR (Figure 4(c)). On the other hand, following exposure to LPS, the total level of PKR was higher in macrophages from middle-aged mice than in those from young mice (Figure 4(b)), although the level of phosphorylation of PKR was lower in macrophages from middle-aged mice than in those from young mice (Figure 4(c)). Macrophages from middle-aged mice also contained more PERK, the other kinase for eIF-2*α*, than those from young mice in the absence of LPS (Figure 5(b)).

### 3.4. Pharmacological Inhibition of eIF-2*α* Dephosphorylation Suppresses the LPS-Stimulated Inflammatory Responses in RAW264.7 Cells

**Figure 6:**
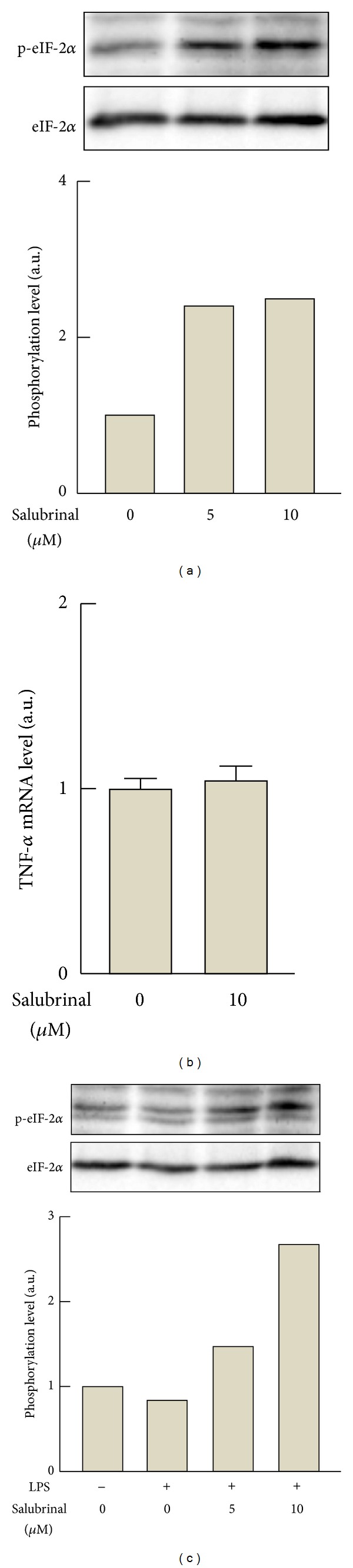
Effects of salubrinal on the phosphorylation of eIF-2*α* in RAW264.7 cells. (a) The cells were treated with 0 (DMSO alone), 5, and 10 *μ*M salubrinal for 6 h. Phosphorylation of eIF-2*α* was analyzed by Western blotting. (b) The cells were stimulated with 10 *μ*M salubrinal or DMSO alone for 6 h. Total RNA extracted from macrophages was converted to cDNA to determine the levels of TNF-*α* mRNA by PCR. The values are corrected with the levels of GAPDH mRNA. Values: means ± SEM (*n* = 3). (c) The cells were stimulated with 100 ng/mL LPS in the presence of 10 *μ*M salubrinal or DMSO alone for 6 h. Phosphorylation of eIF-2*α* was analyzed by Western blotting.

**Figure 7:**
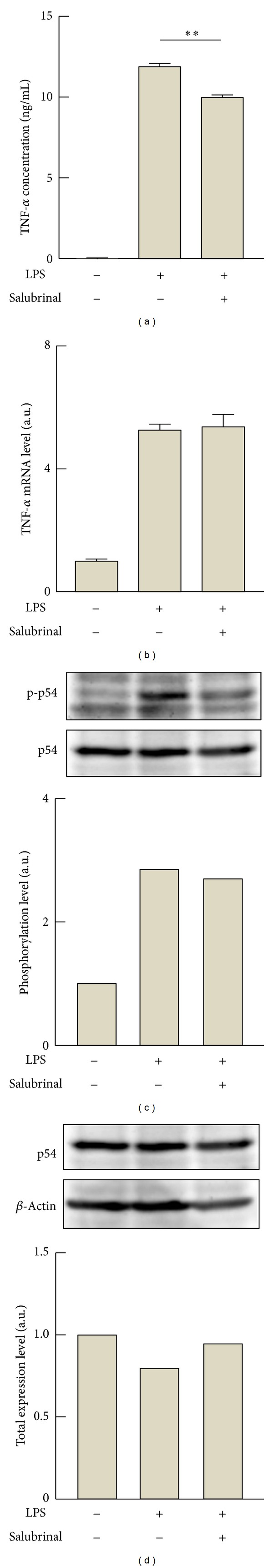
Effects of salubrinal on the LPS-stimulated inflammatory responses of RAW264.7 cells. RAW264.7 cells were stimulated with 100 ng/mL LPS in the presence of 10 *μ*M salubrinal or DMSO alone for 6 h. (a) Concentrations of TNF-*α* in the culture supernatants, measured by ELISA. Values: means ± SEM (*n* = 3). **P < 0.01. (b) Total RNA from macrophages was converted to cDNA to determine the levels of TNF-*α* mRNA by PCR. Values: means ± SEM (*n* = 3). (c) Phosphorylation of the p54 subunit of JNK and (d) abundances of the p54 subunit of JNK were analyzed by Western blotting. Values are shown as means from duplicate samples.

To elucidate a functional role for the inactive form of eIF-2*α* in LPS-stimulated inflammatory responses, the effects of salubrinal on the LPS-stimulated TNF-*α* productions and intracellular signal transductions were examined in RAW264.7 cells. Salubrinal caused an increase in phosphorylation of eIF-2*α* either in the absence of LPS (Figure 6(a)) or in the presence of LPS (Figure 6(c)), and the expression level of TNF-**α** mRNA was not affected by salubrinal in the same experimental condition (Figure 6(b)). The secretions of TNF-*α* from the cells were also not affected by the inhibitor (data not shown). The treatment of the macrophages with salubrinal caused a significant suppression of the LPS-stimulated secretion of TNF-*α* (Figure 7(a)). However, treatment with salubrinal did not affect LPS-induced increase in the abundance of TNF-*α* mRNA (Figure 7(b)). Moreover, LPS-stimulated phosphorylation of JNK p54 subunit was similarly evoked in response to LPS regardless of the presence of salubrinal (Figures 7(c) and 7(d)).

## 4. Discussion

Molecular mechanisms of the attenuated macrophage inflammatory responses against pathogenic factors have been explored mainly in advanced-aged mice [1–13]. These studies focused on age-related reductions in the expression levels of TLR isoforms, their coreceptors such as CD14, and downstream signal proteins such as MAPK and JNK [9–13]. For instance, the mRNA expression levels of all TLR isoforms in splenic and thioglycollate-elicited peritoneal macrophages from 18–24-month-old C57BL/6 mice were significantly lower than those from their 2-3-month-old counterparts [30]. In addition, levels of CD14 on the surfaces of peritoneal and splenic macrophages were lower in advanced-aged BALB/c and C57BL/6 mice than in their younger counterparts [9, 10]. Notwithstanding earlier reports of the age dependency of reduced rates of CD14 expression and LPS-stimulated TNF-*α* secretion [8, 9], the details of the intracellular molecular events in macrophages from middle-aged mice have not yet been fully elucidated.

In this study, we first demonstrated that levels of LPS-stimulated TNF-*α* secretion of resident peritoneal macrophages from middle-aged mice were lower than those from young mice. This result is consistent with previously reported findings [8, 9, 31]. However, we also demonstrated that the expression levels of TNF-*α* mRNA in the cells from middle-aged were increased in response to LPS more greatly than in the cells from young mice. These results suggest that the LPS-stimulated macrophage inflammatory responses are attenuated at the posttranscriptional level in middle-aged mice.

This speculation is supported by our additional findings that the magnitudes of LPS-stimulated increase in JNK and NF-*κ*B signaling in macrophages were comparable in middle-aged and young mice. The abundances and levels of LPS-stimulated phosphorylation of the p54 and p46 subunits of JNK, as well as LPS-stimulated activation of NF-*κ*B, were all lower in advanced-aged BALB/c mice than in their young counterparts [11, 12]. Our findings, along with previously reported observations, suggest that aging is associated with a gradual reduction in the level of LPS-stimulated production of proinflammatory mediators by macrophages, although it is conceivable that the mechanistic basis of the age-related changes is different between advanced-aged and middle-aged mice.

Reduced overall rate of protein synthesis is one of the most common age-related biochemical changes [14, 15]. The rate of protein synthesis is partially regulated by the activities of members of the eIF protein family. For instance, phosphorylation of eIF-2*α* at Ser51 reduces mRNA translation [17, 18]. We observed higher levels of phosphorylation at Ser51 of eIF-2*α* in the macrophages from middle-aged mice than in macrophages from young mice. Moreover, our pharmacological experiments that involved the eIF-2*α* phosphatase inhibitor salubrinal demonstrated that increased phosphorylation of eIF-2*α* prevents LPS-stimulated TNF-*α* secretion without suppressing the induction of TNF-*α* mRNA and TLR4 signaling. These results indicate a functional role for increased phosphorylation of eIF-2*α* in the age-related attenuation of macrophage inflammatory responses.

On the other hand, there were no significant differences in the expression level of ATF4 mRNA between middle-aged and young mice. Our* in vitro* study demonstrated that the expression level of ATF4 mRNA relatively increased approximately 1.1-fold in RAW264.7 cells when eIF-2*α* phosphorylation in the cells increased 2.0-fold by treatment with 10 *μ*M salubrinal for 6 h (data not shown). Therefore, it is conceivable that the increased phosphorylation of eIF-2*α* in middle-aged mice is moderate and does not reach a minimum threshold of its transcriptional activation. Moreover, the suppressive effects of salubrinal on the LPS-stimulated TNF-*α* secretion are relatively lower than those observed in macrophages from middle-aged mice. From these results, the expressions of other eIF subunits and/or the other regulatory systems responsible for mRNA translation such as mammalian target of rapamycin (mTOR) pathway can also be impaired during aging [14, 15].

The total abundance and overall level of phosphorylation of PKR, an eIF-2*α* kinase, were higher in the macrophages from middle-aged mice than in those from young mice. These findings are consistent with a previous report of age-related upregulation of PKR expression and eIF-2*α* phosphorylation in a variety of tissues, which proposed that these changes might be adaptive responses to age-related cellular stresses, including oxidative stress [19]. Accordingly, activation of the PKR gene promoter by H_2_O_2_ treatment was demonstrated in Jurkat T-lymphocytes [32]. Future studies might clarify the effects of oxidative stress on the PKR/eIF-2*α* pathway and determine whether antioxidants can improve the age-related activation of this pathway and the associated attenuation of macrophage inflammatory responses. In addition, the total abundance of PERK protein showed similar tendency to increase as compared to that of PKR, suggesting that the increased phosphorylation of eIF-2*α* during aging may be mediated by functional modulation of endoplasmic reticulum.

The observation that exposure to LPS increased the abundance of the phosphorylated form of PKR in macrophages from young mice and decreased the level of phosphorylation of eIF-2*α* indicated that LPS activates factors besides PKR, such as eIF-2*α* phosphatases. Furthermore, the reduced level of LPS-induced phosphorylation of PKR in macrophages from middle-aged mice compared with young mice suggests age-related deterioration in the robustness of LPS-stimulated activation of such pathways.

In conclusion, LPS-stimulated macrophage inflammatory responses are suppressed at the posttranscriptional level in middle-aged mice. Increased rates of eIF-2*α* phosphorylation might contribute to the age-related attenuation of macrophage inflammatory responses.

## Figures and Tables

**Table 1:** Primers used in the RT-PCR.

Gene	Forward primer sequence	Reverse primer sequence	Product size (bp)	Cycle number
*TNF-*α**	5′-GGA GGG AGA ACA GAA ACT CCA GAA-3′	5′-ATG AGA GGG AGG CCA TTT GGG AA-3′	339	27
*ATF4 *	5′-ATG AGC TTC CTG AAC AGC GAA GTG-3′	5′-GAA CAG GGG AAA GGC TGC AAG AAT-3′	485	30
*GAPDH *	5′-CAG TAT GAC TCC ACT CAC GGC AAA-3′	5′-CAG TGA TGG CAT GGA CTG TGG T-3′	406	22
*18S rRNA *	5′-TGG TGC ATG GCC GTT CTT AGT T-3′	5′-GCA AGC TTA TGA CCC GCA CTT ACT-3′	346	22
